# How SARS-CoV-2 Infection Impacts the Management of Patients with Vulvar Cancer: Experience in a Third-Level Hospital of Southern Italy

**DOI:** 10.3390/jpm13020240

**Published:** 2023-01-29

**Authors:** Luigi Della Corte, Valeria Cafasso, Dominga Boccia, Ilaria Morra, Carmine De Angelis, Sabino De Placido, Pierluigi Giampaolino, Costantino Di Carlo, Giuseppe Bifulco

**Affiliations:** 1 Department of Neuroscience, Reproductive Sciences and Dentistry, School of Medicine, University of Naples Federico II, 84014 Naples, Italy; 2 Department of Public Health, School of Medicine, University of Naples Federico II, 84014 Napoli, Italy; 3 Department of Clinical Medicine and Surgery, University of Naples Federico II, 84014 Naples, Italy

**Keywords:** vulvar cancer, COVID-19, management

## Abstract

**Background:** Since February 2020, the spread of Coronavirus Disease 2019 (COVID-19) in Italy has induced the government to call for lockdown of any activity apart from primary needs, and changing the lives of each of us. All that has dramatically impacted the management of patients affected by cancer. Patients with vulvar cancer (VC) represent a particularly frail population because they are elderly and affected by multiple comorbidities. The aim of this study is to evaluate the clinical impact of the SARS-CoV-2 infection on VC patients in terms of delay or impossibility of carrying out the scheduled treatment. **Methods:** The medical records of patients affected by vulvar tumors, referred to “DAI Materno-Infantile” of AOU Federico II of Naples between February 2020 and January 2022 were retrospectively analyzed. The presence of a positive reverse transcription-polymerase chain reaction (RT-PCR) in nasopharyngeal swab defined the positivity to SARS-CoV-2. **Results:** Twenty-four patients with VC were analyzed and scheduled for treatment. The median age was 70.7 years (range: 59–80). Seven (29.2%) patients were diagnosed with SARS-CoV-2 infection: In three (42.8%) patients, the treatment was delayed with no apparent consequences, in four (57.2%), the treatment was delayed or changed due to cancer progression and, of these four, one died due to respiratory complications of COVID-19, and one died due to oncologic disease progression. **Conclusion:** COVID-19 caused, in most cases, significant delays in oncologic treatments and high mortality in our series of patients affected by VC.

## 1. Introduction

SARS-CoV-2 (severe acute respiratory syndrome coronavirus 2) is an enveloped, positive-sense single-stranded genomic RNA virus (+ssRNA) that belongs to the Betacoronavirus genus [1]. SARS-CoV-2 is the cause of coronavirus disease 2019 (COVID-19), which can produce fatal respiratory tract infections and other serious clinical systemic manifestations. Indeed, the severity of this disease can range from asymptomatic disease or flu-like symptoms to acute respiratory distress syndrome (ARDS), which can lead to death in the most critical cases. Indeed, about 10–15% of the infected people can develop a severe form of COVID-19, mainly in subjects with comorbidities, which may include pneumonia and ARDS, pulmonary embolism, cardiomyopathy, disseminated intravascular coagulation, that may lead to multiple organ failures, and finally death [2]. The first case of SARS-CoV-2 infection was registered in the Wuhan City of China, in December 2019. Meanwhile, in Italy, it was detected for the first time in January 2020. The World Health Organization (WHO) declared a state of emergency due to the increased infection rate, and COVID-19 was defined as pandemic on 11 March 2020. To date, about 418 million confirmed cases and nearly six million deaths due to COVID-19 have been reported worldwide (https://covid19.who.int/, accessed on 18 February 2022). High rates of hospitalization and early mortality in cancer patients have been reported from recent observational studies: The SARS-CoV-2 pandemic has severely and often negatively influenced the management of women with neoplastic disease. In fact, it has been affecting day-to-day cancer care because healthcare policymakers prioritized resource allocation to assist COVID-19 patients. Providing care to neoplastic patients has become extremely challenging, including a reduction in cancer screenings and diagnoses [3,4]. Many medical facilities have decreased access to routine visits to reduce the infection risk for such frail category of women, and bringing significant changes in oncologic patient management, mainly resulting in a delay in the administration of the necessary therapy in a timely fashion, which may particularly affect their long-term survival [5,6,7]. Actually, cancer patients have an increased risk of contracting SARS-CoV-2 infection, developing severe disease, and dying from COVID-19 [8,9,10].

Vulvar squamous cell cancer (VSCC) is an uncommon gynecologic malignant tumor, representing 2–5% of cancers of the female genital tract [11], with an incidence of 2.6 per 100,000 women per year [12]. Squamous cell carcinoma is the most frequent subtype, with a median age of 68 at diagnosis. Vulvar cancer (VC) patients delineate a typical frail population because they are often elderly as well as suffering from multiple comorbidities. The prognosis of the VSCC is poor in case of delayed oncological treatment. It is important to treat these patients in tertiary oncological centers, considering the need for a multidisciplinary group as well as cutting-edge surgical procedures for staging and treatment, such as the groin sentinel node biopsy and, in some cases, the pelvic lymphadenectomy [13,14]. Certain recommendations on the management and surveillance of neoplastic patients during the pandemic have been reported, including for gynecological cancers [15,16,17,18].

Some recommendations about VC management during the COVID-19 pandemic have been proposed, focusing on the type of treatment according to FIGO stage of disease beyond the patient’s clinical characteristics [10].

The aim of this series was to evaluate the clinical impact of SARS-CoV-2 infection among patients with VC planned to the oncologic treatment in terms of delay or impossibility of performing the scheduled treatment.

## 2. Materials and Methods

Data on patients with a diagnosis of active vulvar neoplasm referred to “DAI Materno-Infantile” of AOU Federico II of Naples between March 2020 and January 2022 were collected from the medical records of our Multidisciplinary Cancer Team and retrospectively analyzed. 

The Multidisciplinary Oncology Group (also known as GOM) of our institution is structured in a core team that includes team members for seven central specialties, such as gynecologic oncology, radiation oncology, medical oncology, radiology, and pathology, plus a group of support specialists. All VC cases were reviewed in a weekly meeting, recorded, and handled by three coordinators (L.D.C., I.M., G.B.). Clinical data were carefully collected by V.C. and D.B.

We included in our analysis all patients with SARS-CoV-2 infection between the cancer diagnosis and the start of oncologic treatment or within 30 days after the end of treatment. Obviously, the presence of a positive reverse transcription-polymerase chain reaction (RT-PCR) in nasopharyngeal swab defined the patient as positive for SARS-CoV-2. In our hospital, a qualitative analysis was conducted, considering as positive the presence of SARS-CoV-2 RNA in the sample.

According to our institution protocol, women scheduled for elective surgery underwent a nasal antigen test before pre-anesthetic assessment, RT-PCR detection of SARS-CoV-2 in nasopharyngeal swab within 48–72 h before hospital admission, and a further RT-PCR swab every 5 days until hospital discharge. The personnel assigned detected the body temperature and collected anamnestic data, such as fever in the previous 15 days, cough, ageusia, anosmia, recent contact with positive subjects, because it was required before any outpatient services. 

In case of positive antigenic test, signs, or symptoms suspicious for COVID-19 before an outpatient procedure or before hospitalization, the patients were not accepted, advised to self-isolate, and referred to a general practitioner. If a hospitalized patient turned out to be positive in RT-PCR nasopharyngeal swab, they were transferred to a COVID-19 ward and managed accordingly. 

Patients were considered as recovered when they had molecular swab negativity, and the infection-related symptoms were discontinued.

All data regarding demographic characteristics, histology of VC, scheduled or provided oncologic treatment, delays in treatment, SARS-CoV-2 care setting, infection-related outcomes, and oncologic outcomes were collected and analyzed. 

## 3. Results

Twenty-four patients affected by primary or recurrent vulvar tumors, including Paget disease, were treated in our Institution. Seven out of 24 patients were analyzed because they were simultaneously affected by SARS-CoV-2 infection. The median age was 70.7 years (range 59–80). Surgery for five (71.4%) patients, concomitant chemoradiotherapy for one (14.3%) patient, and chemotherapy for one (14.3%) patient were the planned treatments (Table 1). The stage of disease according to FIGO classification is also reported in Table 1 [19]. The imaging for the proper staging included abdomen and pelvis MRI with contrast medium plus PET/CT total body with 18F-FDG, as for all patients that arrive at our institution.

Five (71.4%) patients were diagnosed with SARS-CoV-2 infection before and two cases (28.6%) after the planned treatment, four patients were vaccinated with two doses, three patients with three doses, and one was not vaccinated. Table 2 reports the summary of these data.

Patient 1, aged 80, had a local recurrence of squamous VC, diagnosed through abdomen and pelvis MRI plus chest CT scan, and was diagnosed with SARS-CoV-2 infection before the planned surgery: After the onset of suspected lung symptoms (one month later), she was hospitalized at another facility for respiratory insufficiency and a diagnosis of interstitial pneumonia was made through chest CT scan. After discharge from the COVID ward, two months later, she underwent further radiologic imaging that showed lymph node and pulmonary disease, so the planned treatment was not administered. She underwent rescue chemotherapy after a negative RT-PCR nasopharyngeal swab, but she died of disease after three months from the diagnosis of tumor recurrence. 

Patient 2, aged 74, was scheduled for elective surgery for primary squamous VC, but COVID-19 infection was discovered after a positive nasal antigen test before acceptance to pre-anesthetic assessment services. The patient complained of anosmia and ageusia without serious signs or symptoms of respiratory infection and was managed at home. No apparent consequences were noted, although the treatment was delayed by three weeks.

Patient 3, aged 72, was scheduled for chemotherapy for systemic recurrence of squamous VC and was diagnosed with SARS-CoV-2 infection after a positive nasal antigenic test before acceptance to pre-anesthetic assessment services. Due to the onset of symptoms, she was managed at home, and, for this reason, the planned drug therapy was delayed by six weeks. Due to a severe worsening of performance status, she was referred to palliative care and died of disease after four months from the oncological. 

Patients 4 and 5, aged 70 and 67, respectively, underwent surgery for primary VC and were discharged on post-operative day 6. The presence of inguinal lymph node metastasis at definitive histological examination (Stage III—T1bN1aM0 according to AJCC TNM 2022) required adjuvant therapy, but because of SARS-CoV-2 infection 30 days after surgery, the first one died of respiratory insufficiency due to interstitial pneumonia, while the second one remained positive for 10 days and then started the therapy without any delay or consequence for her health.

Patient 6, aged 73, affected by locally advanced squamous VC, diagnosed as for Patient 1, was diagnosed with SARS-CoV-2 infection before starting primary chemoradiation and hospitalized due to severe respiratory infection. Her oncological condition worsened (lung metastases), requiring the addition of paclitaxel to cisplatin because of five weeks delay in scheduled treatment. Patient 7, aged 59, affected by vulvar intraepithelial neoplasia (VIN) 3 with focal areas of carcinoma in situ diagnosed by biopsy in September 2021, underwent wide excision only three months later the first diagnosis, fortunately as scheduled for surgery, because of the SARS-CoV-2 infection.

No patient required antivirals for the treatment of SARS-CoV-2 infection.

## 4. Discussion

Oncological patients have an important risk of contracting virus infection and are more likely to have severe COVID-19 and be hospitalized than healthy women due to the immunosuppressed status related to both cancer and drug anticancer therapies, such as chemo and radiotherapy or surgery [8,9,10].

A multicenter, retrospective, cohort study conducted in Wuhan reports how cancer patients with SARS-CoV-2 infection, mainly in the case of advanced tumor stage, manifested a more severe COVID-19 compared with healthy women [17]. Furthermore, patients with hematological malignancies are more likely to develop severe illness than patients with solid organ cancers, besides an increased risk of death during COVID-19-associated hospital recovering [20,21,22]. Moreover, patients with lung cancer or another type of cancer with lung metastasis show the highest risk levels of developing a severe form of COVID-19 compared with healthy women and a relatively high death rate, as well as patients with metastatic cancer (stage IV) [22].

Regarding the effects of COVID-19 on oncologic treatments, delayed surgical/medical treatment, or in some cases, avoided/stopped medical treatment or changes in the surgical plan are unfortunately reported. Moreover, this delay has often been responsible for local or systemic disease progression as well as the deterioration of general condition [5,6,7,23]. The pandemic has led to a reduction in screening, diagnoses, and treatments [23]. Indeed, between March and April 2020, 38.7% of patients with gynecologic cancer in active treatment experienced a change in their health care because of COVID-19, such as delay, change, or cancellation [24].

Recently, Federico et al. reported how this pandemic has caused a significant delay in oncologic treatments and extremely high mortality in VC patients when contracted in the post-operative period, confirming our data [25].

In our series, seven patients were diagnosed with the infection of SARS-CoV-2, particularly, four patients in the pre- and three in the post-treatment period. In three patients (42.8%), the infection led to major clinical implications, three (42.8%) had mild signs or symptoms of respiratory infection, and only one (14.3%) had no symptoms. The median delay in resuming treatment or clinical assessment was about seven weeks. Three (42.8%) patients had the treatment delayed with no apparent consequences since they had no oncologic disease progression. The remaining four (57.2%) had performance status decline: Indeed, two patients died, patient 4 due to SARS-CoV-2 complications and patient 3 due to oncological disease progression and clinical worsening.

There has been an effort worldwide to define strategies for delivering safe cancer surgery during the COVID-19 pandemic, mainly by reorganization of services, such as pre-hospitalization testing for patients with moderate/high risk of COVID-19-infection, restriction of visitors, use of surgical/FFP2-3 mask, physical distancing, and choice of measures to decrease hospitalization. In spite of these measures to contain the infection and limit its spread, cases of SARS-CoV-2 infections acquired in hospitals have been reported consistently. Indeed, the SARS-CoV-2 hospital-acquired infection rate has been reported to be 12–15% [26]. The oncological outcome of cancer patients is based on the type and the timing of treatment. As mentioned above, these patients are vulnerable to COVID-19, and they present increased susceptibility to getting severe infections and complications. Therefore, the widespread strategy of healthcare workers to protect cancer patients from COVID-19 is postponing or canceling clinic visits and treatment according to cancer acuity, to resume the latter when the infection or symptoms have resolved. Unfortunately, this system can result in delayed treatments, which cause possible complications or death of patients [27]. For this reason, it is necessary to evaluate patients individually to highlight any risks deriving from the oncological disease and from the SARS-CoV-2 infection that run along parallel roads but affect each other in order to establish the first treatment to be carried out with the general health of patients as a principal objective and the further steps to be carried out based on the response and evolution of the clinical picture of the individual patient [28].

Another important point concerns whether it could be proper to end anticancer treatment in patients with SARS-CoV-2 infection to resume it when the infection is over. A recent meta-analysis has investigated the impact of anti-tumor approaches on the outcomes of neoplastic patients infected by COVID-19, showing that women with COVID-19 receiving chemotherapy or recently operated might present an increased risk of severe disease or death. In the first case, it is possible to suppose an increased susceptibility to present a bone marrow suppression, such as severe neutropenia or lymphocytopenia, and weakened immunity. Besides, the recovery of the immune system might be slow due to chemotherapy, producing longer and more aggressive viral persistence. In the case of recent surgery, a decreased immunity, clinically manifested, could be activated by stress and trauma related to the surgery [29]. For this reason, it might be appropriate to achieve a shared decision about treatment with the patient after personalized multidisciplinary risk/benefit evaluation. Normally, if a patient has contracted COVID-19, it would be correct to prorogue anticancer treatment until at least 10 days after a positive swab test for SARS-CoV-2 and until any important symptoms have resolved. In any case, it is crucial that cancer is not treated overly to balance the risk of the patient becoming immunosuppressed and developing a serious form of COVID-19 [30].

Regarding radiotherapy, deferring or suspending treatment should be taken into account for patients diagnosed with SARS-CoV-2 infection, as well as increased use of personalized fractionation plans after an evaluation of the expected outcomes of the patient’s clinical conditions.

Concerning patients who are to undergo surgery, some strategies to prevent COVID-19 transmission during hospitalization have been suggested. The most meaningful included: Pre-admission educational and nutritional interventions, avoidance of mechanical bowel preparation, maintenance of normothermia and during surgery, avoidance of surgical drains and nasogastric tubes, removal of urinary catheter on post-operative day 1 in the absence of contraindications [31]. Moreover, careful management of wounds by patients and caregivers and the implementation of outpatient services in the post-operative could perhaps lead to a decrease in hospital stays [26,27].

In the care of patients with VC, the figure of the health personnel is important, especially in this historical period. Physicians, nurses, and other healthcare professionals need to be careful of oncological safety for the correct evaluation of the spread of the disease. Indeed, great progress in the treatment is based on the right pre-, intra-, and post-operative approach that the physician implements based on the patients’ characteristics and clinical picture. An accurate evaluation of the patient’s operability and the extent of surgical resection is necessary, as well as the indication for mapping the sentinel lymph node and possible lymphadenectomy, the setting of post-operative or palliative chemotherapy or radiotherapy.

Based on the latest regulations from the Italian Ministry of Health, COVID-19 vaccination has been suggested to all cancer patients, as well as household contacts and caregivers (https://www.salute.gov.it/portale/nuovocoronavirus/homeNuovoCoronavirus.jsp, accessed on 21 January 2023). Considering the frailty of VC patients and the severe surgical burden associated with the treatment, COVID-19 vaccination has been carried out by all patients included in this study with two doses for patients 1-4 because they were treated before September 2021, when the third dose was not recommended yet for frail patients, and with three doses for patients 5 and 6 treated after that date. Patient 7 did not undergo vaccination despite medical advice. The development of safe and effective vaccines is critical in patients with cancers since they have a higher risk of severe COVID-19. Due to the small number of cases over the years of study, the risks related to the safety of COVID-19 vaccination in cancer patients cannot be estimated. This still represents a major limitation for the population that appears frightened and skeptical. It must be considered that the benefits of vaccination far outweigh the risks of adverse vaccination events as well as COVID-19 infection itself [32,33,34]. However, doubts remain about efficacy in women with active tumors and cancer survivors with chronic immunosuppression due to the variable immune response. These patients, regardless of vaccination, must also implement all the procedures to avoid infection, such as social distancing, the use of a mask, and continuous disinfection [35]. For these reasons, healthcare is giving priority to these patients in terms of just prevention, organization, available personnel, and correct information to mitigate the effects of the pandemic on these particularly vulnerable patients [36].

In our series, the median hospital stay of patients undergoing surgery in the study was 6 days (4–11). Our hospital case history collects about one case per month, and no differences in terms of the number of managed patients were noted between before and after the pandemic, despite the winter peak of SARS-CoV-2 infection.

### Strengths and Limitations

This report points out the impact of SARS-CoV-2 infection in a sample of women with VC, and how it impacts its clinical and surgical management. The main strength is the single institution management of a very rare oncological disease treated with a multidisciplinary approach. The main limitations of this study include the retrospective design and the small number (7) of cases.

## 5. Conclusions

On the whole, this report on the consequences of SARS-CoV-2 infection among patients affected by VC could help to establish the proper management of such condition, generalizing to all cancer patients, and to provide comprehensive data to patients undergoing oncologic treatment in the pandemic era.

## Figures and Tables

**Table 1:** Clinical characteristics of patients.

Patient (N)	Age (Years Old)	Disease (FIGO Stage)	Comorbidities	Planned Treatment	Timing of Diagnosis	Treatment Delayed	Oncologic Outcome	DOD
1	80	Local recurrence of squamous VC (Relapse after Stage I—T1bN0M0)	Obesity	CT (rescue)	Before treatment (community)	3 months	Disease progression, clinical worsening, treatment not administered	No
2	74	Squamous VC (Stage I—T1bN0M0)	Diabetes mellitus	Surgery	Before treatment (pre-anesthetic assessment)	3 weeks	No apparent consequences	No
3	72	Systemic recurrence of squamous VC (Relapse after Stage I—T1bN0M0)	Obesity	Surgery and palliative care	Before treatment (pre-anesthetic assessment)	6 weeks	Worsening of performance status	Yes
4	70	Inguinal lymph node metastasis of squamous VC (Stage III—T1bN1aM0)	None	Surgery	After treatment (community)	-	Disease progression, clinical worsening, treatment not administered.	No
5	67	Squamous VC and inguinal lymph node metastasis (Stage III—T1bN1aM0)	None	Surgery and adjuvant CT	After treatment (community)	10 days	No apparent consequences	No
6	73	Locally advanced squamous VC (Stage III—T1bN1bM0)	None	CT/RT	After treatment (post-operative inpatient care)	5 weeks	Disease progression, addiction to other drugs	No
7	59	Vulvar intraepithelial neoplasia (VIN) 3 with areas of CIS	Obesity	Surgery	Before treatment (community)	3 months	No apparent consequences	No

VC: Vulvar cancer; CIS: Carcinoma in situ; DOD: Dead of disease; CT: Chemotherapy; CT/RT: Chemo-/radiotherapy.

**Table 2:** Characteristics of SARS-CoV-2 infection in patients included in the analysis (ICU: Intensive care unit).

Patient	Symptoms	Care Setting	Complications of SARS-CoV-2 Infection	Death Due to SARS-CoV-2
1	Yes	ICU	Respiratory failure and interstitial pneumonia	No
2	Yes	Home	Anosmia and ageusia without serious signs or symptoms of respiratory infection	No
3	Yes	Home	Mild respiratory symptoms	No
4	Yes	Home	Respiratory failure and interstitial pneumonia	Yes
5	Yes	Home	Mild respiratory symptoms	No
6	Yes	Hospital	Severe respiratory failure	No
7	No	Home	None	No

## Data Availability

Not applicable.

## References

[1] Munster V.J., Koopmans M., Van Doremalen N., Van Riel D., De Wit E. (2020). A Novel Coronavirus Emerging in China—Key Questions for Impact Assessment. N. Engl. J. Med..

[2] Martellucci C.A., Flacco M.E., Cappadona R., Bravi F., Mantovani L., Manzoli L. (2020). SARS-CoV-2 pandemic: An overview. Adv. Biol. Regul..

[3] Patt D., Gordan L., Diaz M., Okon T., Grady L., Harmison M., Markward N., Sullivan M., Peng J., Zhou A. (2020). Impact of COVID-19 on Cancer Care: How the Pandemic Is Delaying Cancer Diagnosis and Treatment for American Seniors. JCO Clin. Cancer Inform..

[4] London J.W., Fazio-Eynullayeva E., Palchuk M.B., Sankey P., McNair C. (2020). Effects of the COVID-19 Pandemic on Cancer-Related Patient Encounters. JCO Clin. Cancer Inform..

[5] Curigliano G., Banerjee S., Cervantes A., Garassino M.C., Garrido P., Girard N., Haanen J., Jordan K., Lordick F., Machiels J.P. (2020). Managing cancer pa-tients during the COVID-19 pandemic: An ESMO multidisciplinary expert consensus. Ann. Oncol..

[6] Eckford R.D., Gaisser A., Arndt V., Baumann M., Kludt E., Mehlis K., Ubels J., Winkler E.C., Weg-Remers S., Schlander M. (2022). The COVID-19 Pandemic and Cancer Patients in Germany: Impact on Treatment, Follow-Up Care and Psychological Burden. Front. Public Health.

[7] Acquati C., Chen T.A., Leal I.M., Connors S.K., Haq A.A., Rogova A., Ramirez S., Reitzel L.R., McNeill L.H. (2021). The Impact of the COVID-19 Pandemic on Cancer Care and Health-Related Quality of Life of Non-Hispanic Black/African American, Hispanic/Latina and Non-Hispanic White Women Diagnosed with Breast Cancer in the U.S.: A Mixed-Methods Study Protocol. Int. J. Environ. Res. Public Health.

[8] Kuderer N.M., Choueiri T.K., Shah D.P., Shyr Y., Rubinstein S.M., Rivera D.R., Shete S., Hsu C.-Y., Desai A., de Lima Lopes G. (2020). Clinical impact of COVID-19 on patients with cancer (CCC19): A cohort study. Lancet.

[9] (2020). COVID-19 More Frequent, Severe in Cancer Patients. Cancer Discov..

[10] Lee K.A., Ma W., Sikavi D.R., Drew D.A., Nguyen L.H., Bowyer R.C.E., Cardoso M.J., Fall T., Freidin M.B., Gomez M. (2020). Cancer and Risk of COVID-19 through a General Community Survey. Oncologist.

[11] Williams E.A., Werth A.J., Sharaf R., Montesion M., Sokol E.S., Pavlick D.C., McLaughlin-Drubin M., Erlich R., Toma H., Williams K.J. (2020). Vulvar Squamous Cell Carcinoma: Comprehensive Genomic Profiling of HPV+ Versus HPV– Forms Reveals Distinct Sets of Potentially Actionable Molecular Targets. JCO Precis. Oncol..

[12] Ramirez P.T., Chiva L., Eriksson A.G.Z., Frumovitz M., Fagotti A., Gonzalez Martin A., Jhingran A., Pareja R. (2020). COVID-19 Global Pandemic: Options for Management of Gynecologic Cancers. Int. J. Gynecol. Cancer.

[13] Garganese G., Fragomeni S.M., Della Corte L., Conte C., Marinucci B., Tagliaferri L., Gentileschi S., Corrado G., Vizzielli G., Scambia G. (2022). Trans-inguinal pelvic lymphadenectomy in vulvar cancer patients: TRIPLE pilot study. Int. J. Gynecol. Cancer.

[14] Garganese G., Inzani F., Fragomeni S.M., Mantovani G., Della Corte L., Piermattei A., Santoro A., Angelico G., Giacò L., Corrado G. (2021). The Vulvar Immunohistochemical Panel (VIP) Project: Molecular Profiles of Vulvar Squamous Cell Carcinoma. Cancers.

[15] Akladios C., Azais H., Ballester M., Bendifallah S., Bolze P.-A., Bourdel N., Bricou A., Canlorbe G., Carcopino X., Chauvet P. (2020). Recommendations for the surgical management of gynecological cancers during the COVID-19 pandemic—FRANCOGYN group for the CNGOF. J. Gynecol. Obstet. Hum. Reprod..

[16] Mancebo G., Solé-Sedeño J.-M., Membrive I., Taus A., Castells M., Serrano L., Carreras R., Miralpeix E. (2020). Gynecologic cancer surveillance in the era of SARS-CoV-2 (COVID-19). Int. J. Gynecol. Cancer.

[17] SEER Cancer Stat Facts: Vulvar Cancer. National Cancer Institute. Bethesda, MD, USA. https://seer.cancer.gov/statfacts/html/vulva.html.

[18] Arpino G., De Angelis C., De Placido P., Pietroluongo E., Formisano L., Bianco R., Fiore G., Montella E., Forestieri V., Lauria R. (2020). Optimising triage procedures for patients with cancer needing active anticancer treatment in the COVID-19 era. ESMO Open..

[19] Pecorelli S. (2009). Revised FIGO staging for carcinoma of the vulva, cervix, and endometrium. Int. J. Gynecol. Obstet..

[20] Tian J., Yuan X., Xiao J., Zhong Q., Yang C., Liu B., Cai Y., Lu Z., Wang J., Wang Y. (2020). Clinical characteristics and risk factors associated with COVID-19 disease severity in patients with cancer in Wuhan, China: A multicentre, retrospective, cohort study. Lancet Oncol..

[21] Lee L.Y.W., Cazier J.-B., Starkey T., Briggs S.E.W., Arnold R., Bisht V., Booth S., Campton N.A., Cheng V.W.T., Collins G. (2020). COVID-19 prevalence and mortality in patients with cancer and the effect of primary tumour subtype and patient demographics: A prospective cohort study. Lancet Oncol..

[22] Dai M., Liu D., Liu M., Zhou F., Li G., Chen Z., Zhang Z., You H., Wu M., Zheng Q. (2020). Patients with cancer appear more vulnerable to SARS-COV-2: A multicenter study during the COVID-19 outbreak. Cancer Discov..

[23] Algera M., van Driel W., Slangen B., Kruitwagen R., Wouters M., Baalbergen A., Cate A.T., Aalders A., van der Kolk A., Kruse A. (2022). Impact of the COVID-19-pandemic on patients with gynecological malignancies undergoing surgery: A Dutch population-based study using data from the ‘Dutch Gynecological Oncology Audit’. Gynecol. Oncol..

[24] Frey M.K., Fowlkes R.K., Badiner N.M., Fishman D., Kanis M., Thomas C., Christos P.J., Martin P., Gamble C., Balogun O.D. (2020). Gynecologic oncology care during the COVID-19 pandemic at three affiliated New York City hospitals. Gynecol. Oncol..

[25] Federico A., Fragomeni S.M., Tagliaferri L., Rios L.S.G., Lancellotta V., Gentileschi S., Corrado G., Gui B., Colloca G., Rufini V. (2021). Clinical impact of SARS-CoV-2 infection among patients with vulvar cancer: The Gemelli Vul.Can multidisciplinary team. Int. J. Gynecol. Cancer.

[26] Glasbey J.C., Nepogodiev D., Simoes J.F.F., Omar O.M., Venn M.L., Evans J.P., Futaba K., Knowles C.H., Minaya-Bravo A., COVIDSurg Collaborative (2020). Outcomes from elective colorectal cancer surgery during the SARS-CoV-2 pandemic. Color. Dis..

[27] Carter B., Collins J., Barlow-Pay F., Rickard F., Bruce E., Verduri A., Quinn T., Mitchell E., Price A., Vilches-Moraga A. (2020). Nosocomial COVID-19 infection: Examining the risk of mortality. The COPE-Nosocomial Study (COVID in Older PEople). J. Hosp. Infect..

[28] Chang A.Y., Cullen M.R., Harrington R.A., Barry M. (2020). The impact of novel coronavirus COVID-19 on noncommunicable disease patients and health systems: A review. J. Intern. Med..

[29] Wu Q., Luo S., Xie X. (2022). The impact of anti-tumor approaches on the outcomes of cancer patients with COVID-19: A meta-analysis based on 52 cohorts incorporating 9231 participants. BMC Cancer.

[30] (2021). COVID-19 Rapid Guideline: Delivery of Systemic Anticancer Treatments.

[31] Thomakos N., Pandraklakis A., Bisch S.P., Rodolakis A., Nelson G. (2020). ERAS protocols in gynecologic oncology during COVID-19 pandemic. Int. J. Gynecol. Cancer..

[32] Desai A., Gainor J.F., Hegde A., Schram A.M., Curigliano G., Pal S., Liu S.V., Halmos B., Groisberg R., Grande E. (2021). COVID-19 vaccine guidance for patients with cancer participating in oncology clinical trials. Nat. Rev. Clin. Oncol..

[33] Akova M., Unal S. (2021). A randomized, double-blind, placebo-controlled phase III clinical trial to evaluate the efficacy and safety of SARS-CoV-2 vaccine (inactivated, Vero cell): A structured summary of a study protocol for a randomised controlled trial. Trials.

[34] Hwang J.K., Zhang T., Wang A.Z., Li Z. (2021). COVID-19 vaccines for patients with cancer: Benefits likely outweigh risks. J. Hematol. Oncol..

[35] Gundavda M.K., Gundavda K.K. (2021). Cancer or COVID-19? A Review of Recommendations for COVID-19 Vaccination in Cancer Patients. Curr. Treat. Options Oncol..

[36] Corti C., Crimini E., Tarantino P., Pravettoni G., Eggermont A.M., Delaloge S., Curigliano G. (2021). SARS-CoV-2 vaccines for cancer patients: A call to action. Eur. J. Cancer..

